# Toward a Molecular Classification of Colorectal Cancer: The Role of BRAF

**DOI:** 10.3389/fonc.2013.00281

**Published:** 2013-11-15

**Authors:** Alexandra Thiel, Ari Ristimäki

**Affiliations:** ^1^Division of Pathology, HUSLAB and Haartman Institute, Helsinki University Central Hospital, University of Helsinki, Helsinki, Finland; ^2^Genome-Scale Biology, Research Programs Unit, University of Helsinki, Helsinki, Finland

**Keywords:** BRAF, colorectal cancer, Lynch syndrome, microsatellite instability, V600E, V600K, vemurafenib, dabrafenib

## Abstract

Different genetic aberrations of *BRAF* have been reported in various malignancies. BRAF is member of the RAS/RAF/MEK/ERK pathway and constitutive activity of this pathway can lead to increased cellular growth, invasion, and metastasis. The most common activating *BRAF* mutation in colorectal cancer is the V600E mutation, which is present in 5–15% of all tumors, and up to 80% of tumors with high microsatellite instability (MSI) harbor this mutation. *BRAF* mutation is associated with proximal location, higher age, female gender, MSI-H, high grade, and mucinous histology, and is a marker of poor prognosis in colorectal cancer. The role of *BRAF* mutation as a predictive marker in respect of EGFR targeted treatments is controversial. *BRAF* V600 selective inhibitors have been approved for the treatment of V600 mutation positive metastatic melanoma, but the response rates in colorectal cancer are poor. This might be due to innate resistance mechanisms of colorectal cancers against the treatment solely targeting BRAF. To overcome resistance the combination of treatments, simultaneous inhibition of BRAF and MEK or PI3K/mTOR, might emerge as a successful therapeutic concept.

## Introduction

BRAF (v-raf murine sarcoma viral oncogene homolog B1) is a serine/threonine protein kinase of the RAF family. RAF proteins are kinases in RAS/RAF/MEK/ERK pathway. ARAF and CRAF are other family members of the RAF family, however, BRAF displays the best binding to RAS and has the highest phosphorylating activity (1). The RAS/RAF/MEK/ERK pathway usually responds to growth factors and cytokines. However, aberrant signaling of this pathway, for example by constantly active kinases can result in abnormal cellular growth, invasion, and metastasis (2).

*BRAF* is mutated at a high frequency in several cancers, although also amplification of the protein and aberrant splicing variants have been reported as well (1). The *BRAF* V600E mutation, deriving from a point mutation of the DNA (1799T → A) is the most common *BRAF* mutation and accounts for around 90% (3). *BRAF* V600E mutation is most prominent in melanoma (40–60%), papillary thyroid carcinoma (45%), low grade serous ovarian carcinoma (35%), and in colorectal adenocarcinoma (5–15%) (4). Other *BRAF* mutations include V600K and V600D/R, accounting for 16–29% and 3% of all *BRAF* mutations in melanoma, respectively (5, 6). Another activating *BRAF* mutation that is almost exclusively found in pilocytic astrocytomas is the *KIAA1549-BRAF* fusion, found in 66–100% of these tumors (7, 8).

Colorectal cancer development and progression can be divided into two separate pathways: chromosomal instability pathway and microsatellite instability (MSI) pathway. In roughly 75% of the cases, colorectal cancer develops through chromosomal instability pathway, and these tumors can harbor *APC* mutations (>90%), *KRAS* mutations (50%), *TP53* mutations (70%), and allelic loss of 18q (80%) (9). MSI pathway covers approximately 15% of sporadic colorectal cancers and almost all Lynch syndrome (LS) cases. In cancers developing through the MSI pathway the DNA mismatch repair (MMR) function is dysfunctional, which leads to insertions and/or deletions of nucleotide repeats in the DNA (9). Remaining tumors belong to CpG island methylator pathway (CIMP) and Serrated Adenoma Pathway, and approximately one third of CIMP tumors are MSI-H while most of the serrated tumors have a deficient *MLH1* gene due to promoter methylation.

## Detection of *BRAF* Mutation in Colorectal Cancer

**Figure 1:**
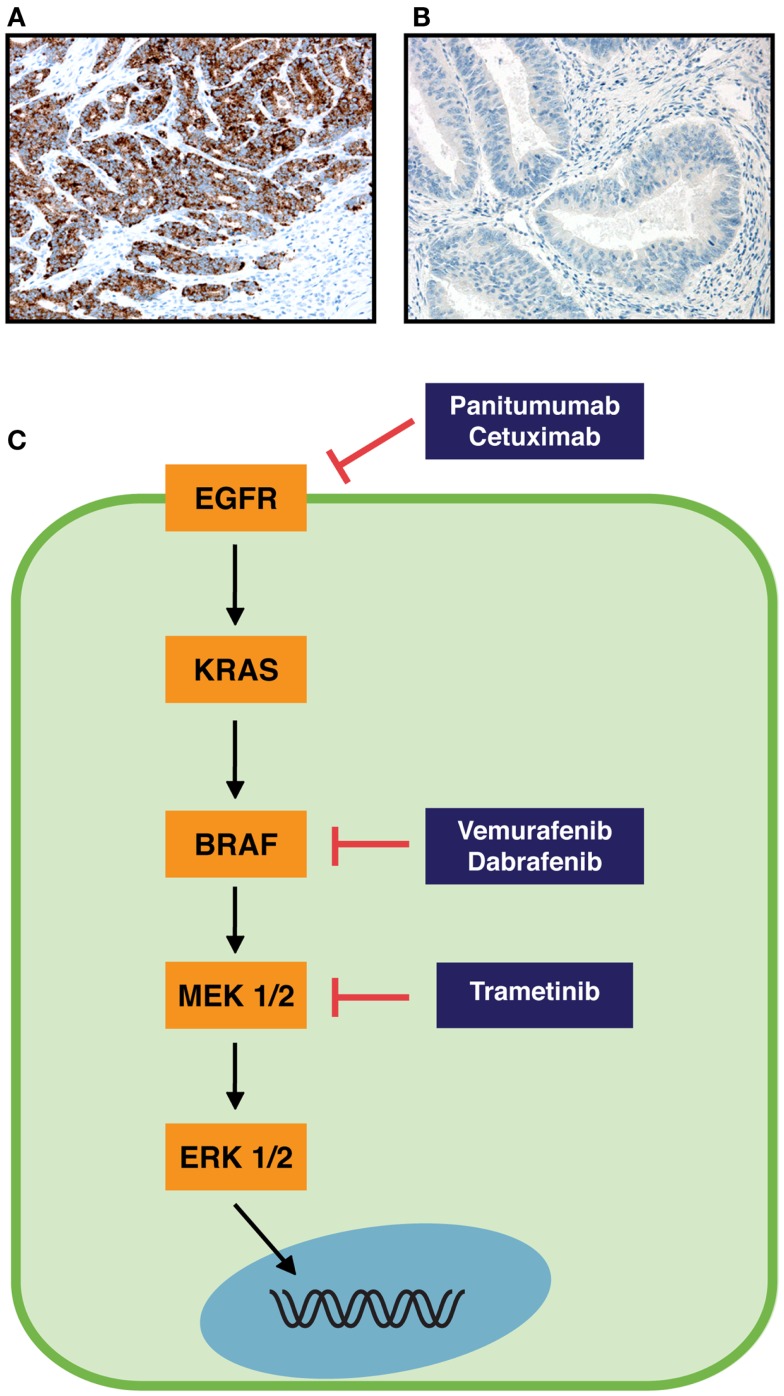
**RAS-RAF pathway and immunohistochemical staining of colorectal cancer specimens with BRAF V600E mutation specific monoclonal antibody**. **(A)** Strong immunopositivity in cancer cells with a BRAF V600E mutation. **(B)** No staining of cancer cells in a specimen without BRAF V600E mutation. Original magnifications are 200×. **(C)** Schematic RAS-RAF pathway (orange boxes) and inhibitors of components of this pathway (blue boxes). Arrows indicate an activation process, and blocked arrows an inhibition process.

Until recently the detection of *BRAF* mutations was performed with Sanger sequencing or PCR-based assays. These methods require representative amount of malignant cells and extraction of the DNA. For specimens with a low content of tumor tissue, the DNA based protocols thus might not be sensitive enough to detect the *BRAF* mutations. A recent report compared the detection of *BRAF* mutations between two next generation sequencing (NGS) technologies and Sanger sequencing/q-PCR and found NGS to be reliable in detecting *BRAF* mutations and other standard-of-care mutations (10). Immunohistochemical (IHC) detection of BRAF V600E with a mutation specific antibody (clone VE1) was first described in metastatic melanoma and papillary thyroid carcinoma (11), and the antibody is currently commercially available (Figures 1A,B). The advantage of IHC lies in the minimal amount of the needed tissue and the availability of this technique in most pathological laboratories. Colorectal cancer has been analyzed with the BRAF V600E mutation specific antibody and most studies find high sensitivities and specificities (98.8–100%) in comparison with PCR-based methods or sequencing (12–16). In one study however, the sensitivity and specificity were only 71 and 74%, respectively (17). The choice of the positive control tissue and the amplification protocol seem to be crucial in successful detection of BRAF V600E mutation by IHC (16).

## Occurrence of *BRAF* Mutation in Colorectal Cancer

The frequency of *BRAF* V600E mutation differs in tumors with high MSI (MSI-H) compared to tumors that are microsatellite-stable (MSS). Whereas *BRAF* V600E mutation frequencies below 10% are reported for MSS tumors (3, 15, 16, 18), they range from 13 to 78% in MSI-H tumors, including cases with germ line mutation for one of the MMR genes (12, 15, 16, 18). In our consecutive colorectal cancer material *BRAF* V600E mutation was found in 78% of MSI-H and 8% of MSS tumors (*p* < 0.0001) (16). A recent study reported *BRAF* V600E mutation in 100% of sessile serrated adenomas/polyps, 94% of traditional serrated adenoma, and in 62% of micro vesicular hyperplastic polyps (19). *BRAF* V600E mutation in microvesicular hyperplastic polyps might indicate the polyps that have a higher risk for progression to adenomas/adenocarcinomas (19). The *BRAF* V600K mutation seems to be a rare event in colorectal cancer, at least in MSI-H tumors (16).

## Significance of BRAF Mutation in Colorectal Cancer

### Connection to clinicopathological parameters

*BRAF* V600E mutations are associated with several clinicopathological parameters and the ones most often reported are: proximal location, higher age, female gender, MSI-H, high grade, and mucinous histology (16, 20–26). Whereas in most studies colorectal cancers are classified into proximal and distal location, Yamauchi et al. described a gradual linear increase of *BRAF* mutation, MSI-H, and high CpG island methylator phenotype frequency from rectum to ascending colon (27). The frequencies of all three factors were lower in cecum than in ascending colon, indicating that cecal cancers are a unique subtype (27).

High microsatellite instability is associated with a higher number of harvested lymph nodes (28, 29), and a recent study reported that *BRAF* V600E mutation was associated with a lower node harvest in the MSI-H group in colon cancer (30). The lymph node count is a predictor of long-term survival in colorectal cancer. Rather than just reflecting the quality of care, the lymph node count might be associated with several factors such as tumor location, tumor and host genetics, and immune interaction (30).

### Prognostic role

*BRAF* V600E mutation is associated with reduced survival (overall survival, disease-free survival, or cancer-specific survival) especially in MSS tumors (Table 1) (18, 21, 23, 24, 26, 31, 32). Its role in MSI-H tumors is not so clearly defined; while some studies attribute MSI-H tumors with excellent survival regardless of *BRAF* status (18), *BRAF* V600E mutation decreased overall survival independent of MSI status in another report (25). In addition, *BRAF* V600E mutation was associated with poor prognosis in all groups of advanced colorectal cancer (33) and was an independent prognostic factor for overall survival and cancer-specific survival in a pooled stage II/III cohort (22). In a couple of studies, no prognostic role was found to be associated with *BRAF* mutation (Table 1) (34, 35). Finally, in a meta-analysis that included 26 colorectal cancer studies, *BRAF* mutation was found to increase the risk of mortality (HR = 2.25, 95% CI: 1.82–2.83) (36).

**Table 1:** ***BRAF* mutation as prognostic factor in colorectal cancer**.

*BRAF* mutation as prognostic factor	Tested for *BRAF* mutation (*BRAF* mutated)	Comments	Reference
Independent	911 (87)	Stage II-IV, microsatellite-stable tumors, age, stage, tumor site, and CpG island methylator phenotype adjusted, reduced OS, HR = 3.06, 95% CI: 2.06–4.54; (1.0 reference *BRAF* wt)	Samowitz et al. (18)
Independent	1307 (103)	Stage II/III, reduced OS, HR = 1.78, 95% CI: 1.15–2.76; (1.0 reference *BRAF* wt)	Roth et al. (21)
Independent	297 (59)	Stage II/III, reduced OS, HR = 0.45, 95% CI: 0.25–0.8, and reduced cancer-specific survival, HR = 0.47, 95% CI: 0.22–0.99; (1.0 reference *BRAF* mut)	Farina-Sarasqueta et al. (22)
Independent	506 (75)	Stage III, reduced OS, HR = 1.66; 95% CI: 1.05–2.63; (1.0 reference *BRAF* wt)	Ogino et al. (23)
Independent	475 (56)	Stage I-III, reduced OS, HR = 1.79, 95% CI: 1.05–3.05; (1.0 reference *BRAF* wt)	Kalady et al. (25)
Independent	196 (35)	Stage I-IV, reduced cancer-specific survival, HR = 2.00, 95% CI: 1.16–3.43; (1.0 reference *BRAF* wt)	Eklöf et al. (26)
Independent	1253 (182)	Stage I-IV, higher cancer-specific mortality in microsatellite-stable tumors, HR = 1.60, 95% CI: 1.12–2.28; (1.0 reference MSS/*BRAF* wt)	Lochhead et al. (32)
Non-independent	711 (56)	Advanced CRC, reduced OS, HR = 1.82; 95% CI: 1.36–2.43; (1.0 reference *BRAF* wt)	Richman et al. (33)
Non-independent	181 (20)	Stage I-IV, proficient DNA mismatch repair, stage-adjusted reduced OS and DSF, HR = 6.63, 95% CI: 2.60–16.94 and HR = 6.08, 95% CI: 2.11–17.56; (1.0 reference *KRAS/BRAF* wt)	Pai et al. (24)
Non-independent	243 (18)	Metastatic CRC, reduced PSF, HR = 2.39, 95% CI: 1.36–4.21; (1.0 reference *KRAS/BRAF* wt)	Peeters et al. (31)
No prognostic significance	490 (77)	Stage II/III, no effect on DFS, HR = 1.0, 95% CI: 0.6–1.6; no effect on OS, HR = 1.2, 95% CI: 0.8–1.8; (1.0 reference *BRAF* wt)	French et al. (34)
No prognostic significance	822 (10%)	Stage II/III, no effect on DFS, HR = 1.07, 95% CI: 0.66–1.73; (1.0 reference *BRAF* wt)	Mouradov et al. (35)

*CRC, colorectal cancer; DSF, disease-free survival; mut, mutant; OS, overall survival; PFS, progression-free survival; wt, wild-type*.

### Predictive role

It has been suggested that in order for metastatic colorectal cancer patients to receive a response for treatment with monoclonal antibodies targeting EGFR (panitumumab and cetuximab, Figure 1C), the *BRAF* gene needs to be present as wild-type (37, 38). Yuan et al. recently concluded in a meta-analysis that *BRAF* mutation is a predictive biomarker and indicates poor prognosis when metastatic colorectal cancer patients are treated with monoclonal antibodies against EGFR (39). In contrast to these results, a recent guideline does not recommend testing for *BRAF* mutations in colorectal cancer patients before anti-EGFR treatment (40). Garcia-Alfonso et al. (40) conclude that *BRAF* mutation is not predictive for anti-EGFR treatment in randomized trials. For patients (*KRAS* wild-type metastatic colorectal tumors) treated with chemotherapy/bevacizumab with or without cetuximab in the phase III CAIRO2 study, *BRAF* mutation was correlated to a shorter progression-free survival and overall survival, in both treatment arms (41). Similarly, *BRAF* mutation was not predictive for treatment with cetuximab, but was a marker of poor prognosis in metastatic colorectal cancer patients (*KRAS* wild-type) that were randomly assigned to treatment with FOLFIRI (irinotecan, fluorouracil, leucovorin) with or without cetuximab in the CRYSTAL study (42). The pooled analysis of the CRYSTAL and OPUS trials on metastatic colorectal cancer showed that *BRAF* mutation was not predictive for treatment with cetuximab in *KRAS* wild-type patients, but indicated poor prognosis (43). Finally, in a retrospective analysis of the PRIME study, *BRAF* mutation was not predictive for overall or progression-free survival in *KRAS* wild-type patients treated with FOLFOX4 (oxaliplatin, fluorouracil, leucovorin) with or without panitumumab (44).

As for treatment with standard chemotherapy agents (fluorouracil with irinotecan or oxaliplatin), *BRAF* V600E mutation was not predictive (33). Similarly, *BRAF* mutation was not predictive for fluorouracil-based therapy in mostly stage II colorectal cancer (45). A non-significant trend for better survival with fluorouracil/leucovorin + irinotecan (vs. fluorouracil/leucovorin alone) was detected in colorectal cancer stage III patients with *BRAF* V600E mutation (23).

### Role in identifying LS patients

Lynch syndrome is a hereditary form of colorectal cancer that accounts for 1–3% of all CRC cases. It is the most common form of hereditary CRC and is caused by a germ line mutation of one of the MMR genes (46). As not all LS patients fulfill the Amsterdam II criteria or revised Bethesda guidelines, not all of them are detected in the routine clinical setting (47, 48). *BRAF* is usually present as wild-type in LS patients, and only 1.4% of the LS patients carry a *BRAF* V600E mutation (49). In sporadic colorectal cancer the *BRAF* V600E mutation rate ranges from 5 to 15% (4), and in the MSI-H group of consecutive primary colorectal cancers the *BRAF* V600E mutation rate reached 78% (16). This has led to the suggestion that the detection of *BRAF* V600E mutation might be a useful additional tool in finding LS patients, and several recent studies have used *BRAF* V600E IHC to implement this step (12, 15, 16).

## BRAF Inhibitors in Treatment of Cancer

The first RAF inhibitor, sorafenib, was not effective in clinical use for metastatic melanoma, as it did not improve median overall survival in randomized, double-blind, placebo-controlled phase III studies, when given in combination with paclitaxel and carboplatin as second-line treatment or to chemotherapy-naïve patients (50–52). The reason for the disappointing results with sorafenib in melanoma might be that this multi-targeted tyrosine kinase inhibitor has a higher affinity for isoforms other than BRAF and targets several other pathways as well (50, 53). However, in advanced hepatocellular carcinoma, the median survival time was increased by nearly 3 month in patients treated with sorafenib, in a phase III, double-blind, placebo-controlled trial (54). Vemurafenib (PLX4032) and dabrafenib (GSK2118436) are approved for treatment of unresectable or metastatic melanoma (Food and Drug Administration) and vemurafenib is also approved by the European commission/European Medicines Agency. Both selectively inhibit the *BRAF* V600 mutated form of BRAF, inhibit phosphorylation of ERK, and have high clinical response rates in melanoma patients (Figure 1C) (50, 53). Whereas patients with *BRAF* V600 mutated melanomas had a clear survival benefit when treated with BRAF inhibitors, the response rate in metastatic colorectal carcinoma (harboring *BRAF* V600E mutation) was poor, since only one patient (1/19) displayed a partial response and 4 out of 19 patients a minor response (55, 56). It has been noted already in xenografts from BRAF V600E mutant colorectal cancer cell lines that tumor growth inhibition was most efficient when vemurafenib was combined with EGRF or Akt inhibitors and/or chemotherapeutic agents (57).

## Resistance to BRAF Inhibition in Melanoma and Colorectal Cancer

*BRAF* V600E mutant melanomas initially have a good response rate. However, most of them acquire a drug resistance after 6–7 months, and roughly 10% have tumor progression at earlier stages (53, 55). *BRAF* V600E mutated colorectal cancer on the contrary, seems to display an innate resistance to inhibition with BRAF inhibitors, which was also demonstrated in colorectal cancer cell lines (55, 58, 59). The mechanisms of resistance can be grouped according to their dependence on ERK signaling (60). ERK-dependent resistance mechanisms can occur via activating *MEK1* mutations (61), activating *NRAS* mutations (62), *COT* overexpression (63), elevated CRAS activity (64), *BRAF* V600E alternative splicing or amplification (65). ERK-independent mechanisms include the PI3K pathway (66), overexpression of PDGFRβ (62), IGF1R activation (67), and hepatocyte growth factor (59). Importantly, Romano et al. report that different mechanisms of resistance can occur in the same patient at different metastatic locations (68).

In *BRAF* V600E mutant colorectal cancer cells the amplification of the *BRAF* gene was identified as mechanism of resistance to MEK and BRAF inhibition (69). Two studies detected the critical role of EGFR in *BRAF* V600E mutant colorectal cancer cells that did not respond to BRAF inhibition (58, 70). Corocan et al. reported that *BRAF* V600E mutant colorectal cancer cell lines harbored more phospho-EGRF than melanomas with the same mutation, and reactivated MAPK signaling via EGFR (58). Prahallad et al. described a rapid feedback activation of EGFR (via CDC25C inhibition) upon RAF inhibition, and EGFR was highly expressed *BRAF* V600E mutant colorectal cancer cells as compared to *BRAF* V600E mutant melanoma cells (70).

## Overcoming of Resistance and Combination Treatments

To overcome resistances upon treatment with a BRAF inhibitor, targeting novel downstream kinases of the pathway or combination of therapies might be helpful. As for melanoma treatment, the combination of vemurafenib with the HDM2 inhibitor nutlin-3 (leading to p53 restoration), has shown synergistic effect on inducing apoptosis and suppressing tumor growth in melanoma cell lines and xenografts (71). Novel combinatorial treatment options include BRAF inhibition simultaneously with PI3K/mTOR as shown in colorectal cell lines and animal models (72–74). Coffee et al. used the *Apc-Braf* mouse model (mice bearing a *BrafV600E* allele) and showed that concomitant inhibition of PI3K/mTOR and BRAF resulted in tumor regression due to induction of apoptosis and decrease in proliferation (73). Also Rad et al. reported the potent growth inhibitory effect of combined BRAF/PI3K inhibition on xenografts of *BRAF* mutant mouse and human colorectal cancer cell lines (74). Furthermore, MEK inhibition alone caused regression of xenografted and orthotopically transplanted tumors, and reduced proliferation in tumors of *Braf^LSL-V637E/^*^+^ mice (orthologous to human *BRAF* V600E mutation) (74). A combined inhibition of BRAF (dabrafenib 150 mg) and MEK1/2 (trametinib, 1 or 2 mg) was performed in metastatic melanoma patients with *BRAF* V600E mutation, in a open-label phase II study with randomly assigned patients. Both median progression-free survival (9.4 vs. 5.8 months) and complete/partial response (76 vs. 54%) were significantly improved in the combination group (150 + 2 mg) vs. dabrafenib immunotherapy (75). Both dabrafenib and trametinib, were recently (May 2013) approved by the Food and Drug Administration for treatment of metastatic/inoperable melanoma (Figure 1C).

## Conclusion

*BRAF* V600E mutation is a marker of poor prognosis in colorectal cancer. Detection of this mutation can also be used to identify LS patients. Targeted treatment of *BRAF* V600E mutation is in use in advanced melanoma. However, the response is short-lived in melanoma patients, due to the development of acquired resistance. In colorectal cancer targeted treatment of mutated *BRAF* is not feasible due to the innate resistance. New insights into possible resistance mechanisms were reported recently, and combinatorial treatment options might impact therapy of tumors carrying a *BRAF* mutation.

## Authors’ Note

After acceptance of this review, a novel study reported the combined use of BRAF V600E and MMR immunohistochemistry as a prognostic tool in colorectal cancer (Toon CW, Chou A, DeSilva K, Chan J, Patterson J, Clarkson A, et al. BRAFV600E immunohistochemistry in conjunction with mismatch repair status predicts survival in patients with colorectal cancer. Modern Pathol (2013) Oct 25. doi:10.1038/modpathol.2013.200). The authors restricted their analysis to only immunohistochemistry of BRAF V600E and MMR status on 1426 consecutive colorectal cancer cases, and found that MSS/BRAF V600E mutant tumor status was a marker for poor prognosis in univariate analysis when compared to MSS/BRAF wild type tumors (HR = 1.79, 95% CI: 1.24–2.60). Immunohistochemical screening for BRAF V600E mutation and MMR gene expression thus can facilitate the detection of Lynch syndrome patients and can also identify subgroups with a poor prognosis.

## Conflict of Interest Statement

The authors declare that the research was conducted in the absence of any commercial or financial relationships that could be construed as a potential conflict of interest.
